# Impact of Type 1 Diabetes on Renal Parameters in Children Aged One to 17 at the Mother and Child University Hospital in N’Djamena, Chad

**DOI:** 10.7759/cureus.58082

**Published:** 2024-04-11

**Authors:** Kevin Ganita, Guy Sedar Singor Njateng, Sammer Yousuf

**Affiliations:** 1 Biochemistry, University of Dschang, Dschang, CMR; 2 International Center for Chemical and Biological Sciences, HEJ Research Institute of Chemistry, University of Karachi, Karachi, PAK

**Keywords:** renal parameters, n'djamena-chad, child, nephropathy, diabetes

## Abstract

Background

Diabetes is a metabolic disease caused by a defect in the secretion of insulin or its misuse. It is a major public health problem worldwide. While type 2 diabetes generally affects people of advanced age, type 1 diabetes generally occurs in people of younger ages and its prevalence is increasingly high among children in Chad. When it is poorly managed, it can be accompanied by several functional complications including renal failure. In order to have an overview of the incidence of this complication in children suffering from type 1 diabetes and to contribute to its better follow-up, a study was conducted at the Mother and Child University Hospital in N'Djamena whose objective was to assess the impact of type 1 diabetes on renal parameters in children aged one to 17 years.

Methodology

A cross-sectional study was conducted from April to June 2023 at the Mother and Child University Hospital Center in N'Djamena on 61 children with type 1 diabetes aged one to 17 years. A questionnaire sheet was submitted to the parents of the participants and the data from this sheet were analyzed while biochemical parameters were evaluated using standard commercial kit methods, the IONIX SFRI automated ion analyzer, HemoCue HbA1c501® hemoglobinometer, and spectrophotometer (BioSystems).

Results

Participants were ranked according to glycemic control and duration of diabetes discovery. 73.61% of the children showed alterations in renal parameters, some of which increased and others decreased; 86.9% had poor glycemic control, which is associated with alterations in renal parameters in study participants. Multiple logistic regression showed hypercreatinemia, hyperuremia, hyperglycemia, hyperhemoglobinemia, hyperchloremia, hyponatremia, hypokalemia, hyperglycemia, hyperketonuria, hyperproteinuria, and decreased glomerular filtration rate (GFR).

Conclusion

It appears from this study that type 1 diabetes through glycemic control and the duration of discovery has an increasing impact on certain renal parameters and a decrease in others, leading to impaired renal function.

## Introduction

The World Health Organization (WHO) defines diabetes as a complex metabolic disorder characterized by chronic hyperglycemia accompanied by disturbances in the metabolism of carbohydrates, lipids, and proteins due to defects in the secretion and/or action of insulin [1]. Over half a billion people are living with diabetes worldwide [2]. There are two main types of diabetes: type 1 diabetes, which is the subject of our study, and type 2 diabetes, which represents more than 90% of the cases of diabetes detected worldwide [3]. Type 1 diabetes, on the other hand, accounts for approximately 5-10% of all diabetes cases worldwide and is usually diagnosed in children and young adults [4]. Approximately 1.1 million children and adolescents worldwide live with type 1 diabetes [5].

Previous studies demonstrate that diabetes is associated with a significant increase in mortality, mainly due to its long-term complications. More recently, this excess mortality has been found to be concentrated in the subset of people with diabetes who develop kidney disease, both in type 1 diabetes [6]. Diabetes can be the cause of several complications that are the subject of studies today. It can cause disturbances in kidney parameters leading to serious health problems; it is, therefore, the leading cause of renal failure [7,8].

According to a new WHO analysis, only 55% of people with diabetes know their status globally and only 46% in the African region. This low rate is due to certain obstacles to diabetes screening, namely the lack of facilities and screening equipment, the inadequate number of trained health personnel, insufficient access to health facilities, and the lack of awareness of diabetes [9]. All these difficulties increase the prevalence of the disease as well as these complications, particularly diabetic nephropathy in Africa in general and in Chad in particular [10]. In 2019, diabetes and kidney disease due to diabetes caused approximately two million deaths worldwide yet, in Chad, little research has been carried out in the field of diabetes, resulting in a lack of data and diabetes management strategies [11]. It is on the basis of these observations that this study was carried out in order to bring to light the influence of type 1 diabetes on the renal function of children from one to 17 years old at the Mother and Child University Hospital in N’Djamena, Chad.

## Materials and methods

Design and study population

This study was a cross-sectional study conducted at the Mother and Child University Hospital Center of N'Djamena, Chad, from April to June 2023. The National Ethics Committee validated the study protocol for Human Health in N'Djamena (ref: 025/PT/PM/MESRSI/SEESRSI/SG/2023). The participants of the study were children and adolescents aged one to 17 years without distinction of sex and all type 1 diabetics whose consent and informed consent of the parents were obtained.

Collection of blood samples

A questionnaire sheet was submitted to participants who gave their assent and whose consent was obtained as well and a blood collection of a volume of 5 mL at the bend of the elbow using an alcohol swab and a syringe attached to the needle was done. Also, a collection of urine over 24 hours in well-closed sterile containers was performed. The blood sample was collected in the lithium heparin tube and then centrifuged at 3000 rpm for 5 minutes to obtain the serum. The latter was therefore separated from the plasma in micro tubes for analysis.

Sample analysis

Biochemical examinations including blood sugar, creatinine, and uremia were performed using the spectrophotometric method using the spectrophotometer (BioSystems). Natremia, chloremia, and serum potassium were measured using the IONIX SFRI automated ion analyzer; glycated hemoglobin was quantified by the HemoCue HbA1c501® hemoglobinometer. Glycosuria, ketonuria, and proteinuria were performed by colorimetry using urinary strips (commercial kits).

Statistical analysis

Biochemical data were analyzed using Statistical Software for the Social Sciences (SPSS) version 18 version 22.0. The results were expressed as mean ± standard deviation. The means of each biochemical parameter in different groups were compared using the Waller-Duncan test while significant differences were detected by ANOVA. Waller Duncan's conformity test was used to compare the means of group variables to reference values. The threshold values provided by the kits were used to classify the parameters as abnormal (value outside the reference range) and normal (value within the reference range). Categorical variables were described as numbers and percentages. A chi-square test was performed to compare the frequencies of biochemical abnormalities in the three groups.

## Results

This study focused on 61 type 1 diabetic patients (Table 1) at the Mother and Child University Hospital Center of N'Djamena and those who were newly received in the laboratory and Pediatrics 2 of the said center. Among these patients, 55.7% were male while 44.3% were female and the average age of this study population was 12.85 with a standard deviation of 3.010 years. This population was divided into three age groups, and the age group from 11 to 17 years was the most represented and constituted 85.2% of the population. The majority of participants (36.1%) had a primary education level and lived mostly in urban areas, i.e., 80.3% of the population.

**Table 1:** Distribution of participants according to socio-demographic parameters

Parameters	Category	Number (%)
Sex	Male	34 (55.7)
	Feminine	27 (44.3)
Age range	1-5 years	2 (3.3)
	6-10 years old	7 (11.5)
	11-17 years old	52 (85.2)
Educational level	Not in school	19 (31.1)
	Primary	22 (36.1)
	Secondary	20 (32.8)
Current residence	Urban	49 (80.3)
	Rural	12 (19.7)
Parent function	Unemployed	12 (19.7)
	Informal sector	23 (37.7)
	Official	26 (42.6)

The average fasting blood glucose observed in these patients was 4.54±1.06 mg/dL, with 99.99% of participants in a situation of hyperglycemia (Table 2). Similarly, the average HbA1c for these patients was 10.71% with a standard deviation of 2.73% and the patients were divided into two groups according to their HbA1c level: those with a level greater than 7% (poor glycemic control) representing 86.9% of the population and those with a rate below 7% (good glycemic control) representing 13.1% of the population. Most of the children were of normal weight (67.2%) and the average body mass index (BMI) was 17.06 (±4.41).

**Table 2:** Number and mean of biochemical parameters and BMI GFR, glomerular filtration rate; BMI, body mass index

Parameters	Category	Number (%)	Average
BMI	Less than 14.5 - thinness	17 (27.9)	17.06 (±4.41)
	Between 14.5 and 22 - normal weight	41 (67.2)	
	Between 22 and 26.5 - overweight	2 (3.3)	
	Above 27 - obesity	1 (1.6)	
HbA1c	<7%	8 (13.1%)	10.71 (±2.73)
	>7%	53 (86.9%)	
Creatinine	Normal: 2.4-8.7 mg/L	33 (54.1%)	11.09 (±9.82)
	Hypercreatinemia: >8.7 mg/L	28 (45.9%)	
Urea	Hypouremia: <0.15 g/L	12 (19.7%)	0.36 (±0.30)
	Normal: 0.15-0.38 g/L	23 (37.7%)	
	Hyperuremia: >0.38 g/L	26 (42.6%)	
Sodium ion	Hyponatremia: <135 mmol/L	39 (63.9%)	131.54 (±20.04)
	Normal: 135-145 mmol/L	11 (18%)	
	Hypernatremia: >145 mmol/L	-	
Potassium ion	Hypokalemia: <3.5 g/L	47 (77%)	3.01 (±0.72)
	Normal: 3.5-5.1 g/L	14 (23%)	
	Hyperkalemia: >5.1g/L	-	
Chloride ion	Hypochloremia: <95 mmol/L	3 (4.9%)	118.96 (±22.47)
	Normal: 95-105 mmol/L	17 (27.9%)	
	Hyperchloremia: >105 mmol/L	41 (67.2%)	
Glycosuria	Negative	1(2.6)	10.12 (±5.35)
	A cross (2.5 g/L)	3 (4.9)	
	Two crosses (5 g/L)	14 (23)	
	Three crosses (10 g/L)	32 (52.5)	
	Four crosses (20 g/L)	11 (18)	
Ketonuria	Negative	2 (3.3)	0.48 (±0.30)
	A cross (0.15 g/L)	14 (23)	
	Two crosses (0.4 g/L)	24 (39.3)	
	Three crosses (0.8 g/L)	20 (32.8)	
	Four crosses (1.60 g/L)	1 (1.6)	
Proteinuria	Presence	6 (9.8)	
	Absence	55 (90.2)	
GFR	<15 mL/min/1.73m² RF: terminal	1 (1.6)	75.89 (±33.66)
	15-59 RF: beginning	18 (29.5)	
	>60: normal	42 (68.4)	

For renal parameters, hypercreatinemia was observed in 45.9% (28) of the study population with an average of 11.09 (±9.82) m/L while in most cases (26 children i.e., 42.6%) hyperuremia was observed with an average of 0.36 (±0.30) g/L. Furthermore, 63.9% (i.e., 39) of patients showed hyponatremia with an average of 131.54 (±20.04) mmol /L while hypokalemia was observed in 47 (77%) of the children with an average of 3.01 (±0.72) g/L. Table 2 summarizes the effective mean of the parameters. While the average BMI during this study was 17.065±4.417, it appears that 67.2% of the study population had normal weight, and 27.9% of slimming patients, while 3.3% were overweight.

The analysis of the descriptive statistics through the cross tables gives the number of participants by age group for the quantitative parameters studied (Table 3). For the BMI, the most represented age group is that of children aged 11-17 years with 76.47% of lean patients. All patients were in a situation of hyperglycemia and 84.90% had an HbA1c level greater than 7% (hyperglobunemia) in this age group. Hypercreatinemia and hyperuremia in this subpopulation were 82.14% and 92.30%, respectively. For ion assays, 84.61% of children were hyponatremic, 82.97% were hypokalemic, and 82.92% were hyperchloremic.

**Table 3:** Distribution of biochemical abnormalities according to age group RF, renal failure; GFR, glomerular filtration rate; BMI, body mass index

Parameters		Category	Age group Number (%)		
			1-5 years	6-10 years	11-17 years
BMI	Less than 14.5: thinness		1 (50)	3 (42.85)	13 (25)
	Between 14.5 and 22: normal weight		1 (50)	4 (57.15)	36 (69.23)
	Between 22 and 26.5: overweight		0 (0)	0 (0)	2 (3.84)
	Above 27: obesity		0 (0)	0 (0)	1 (1.93)
HbA1c	<7%		1 (50)	0 (0)	7 (13.46)
	>7%		1 (50)	7 (100)	45 (86.53)
Creatinine	Normal: 2.4-8.7 mg/L		1 (50)	3 (42.85)	29 (55.76)
	Hypercreatinemia: >8.7 mg/L		1 (50)	4 (57.15)	23 (44.23)
	Hypouremia: <0.15 g/L		/	/	/
Urea	Normal: 0.15-0.38 g/L		0 (0)	1 (14.28)	11 (21.15)
	Hyperuremia: >0.38 g/L		2 (100)	4 (57.15)	17 (32.69)
	Hyponatremia: <135 mmol/L		0 (0)	2 (28.57)	24 (48.15)
Sodium ion	Normal: 135-145 mmol/L		0 (0)	6 (85.71)	33 (63.46)
	Hypernatremia: >145 mmol/L		1 (50)	1 (14.28)	9 (17.30)
	Hypokalemia: <3.5 g/L		1 (50)	0 (0)	10 (19.23)
Potassium ion	Normal: 3.5-5.1 g/L		1 (50)	7 (100)	39 (75)
	Hyperkalemia: >5.1g/L		1 (50)	0 (0)	13 (25)
Chloride ion	Hypochloremia: <95 mmol/L		0 (0)	0 (0)	3 (5.76)
	Normal: 95-105 mmol/L		0 (0)	2 (28.57)	15 (28.84)
	Hyperchloremia: >105 mmol/L		2 (100)	5 (14.28)	34 (65.38)
Glycosuria	Negative		0 (0)	0 (0)	1 (1.93)
	A cross (2.5 g/L)		0 (0)	0 (0)	3 (5.76)
	Two crosses (5 g/L)		0 (0)	2 (28.57)	12 (23.07)
	Three crosses (10 g/L)		1 (50)	4 (57.15)	27 (51.92)
	Four crosses (20 g/L)		1 (50)	1 (14.28)	9 (17.30)
Ketonuria	Negative		0 (0)	0 (0)	2 (3.84)
	A cross (0.15 g/L)		1 (50)	1 (14.28)	12 (23.07)
	Two crosses (0.4 g/L)		0 (0)	4 (57.15)	20 (38.46)
	Three crosses (0.8 g/L)			2 (28.57)	17 (32.69)
	Four crosses (1.60 g/L)		0 (0)	0 (0)	1 (1.93)
Proteinuria	Presence		0 (0)	1 (14.28)	5 (9.61)
	Absence		2 (100)	6 (85.71)	47 (90.38)
GFR (mL/min/1.73m²)	<15 mL/min/1.73m²: terminal RF		0 (0)	0 (0)	1 (1.93)
	15-59: beginning RF		1 (50)	4 (57.85)	13 (25)
	> 60: normal GFR		1 (50)	3 (42.15)	38 (73.07)

Glycosuria, a diagnostic factor of diabetes, is more represented in children aged 11-17 years. 83.33% and 84.37% of children in this population showed ketonuria and hyperglycosuria, respectively. The number of children with early kidney failure was 18, of which 72.22% were aged 11-17 years. Table 3 links these different parameters to the age group.

Table 4 summarizes the means and the p-values of various parameters influencing the patient's glycemic control but also of the renal markers according to the age groups carried out using the one-factor ANOVA test. According to age group, there is no significant difference between different biochemical parameters.

**Table 4:** Averages of the different biochemical parameters according to age group GFR, glomerular filtration rate; BMI, body mass index

Parameters	Normal range	Age group			P-value	
		1-5 years	6-10 years	11-17 years		
		Mean (standard deviation)				
BMI	14.5-22	15.90 (±4,246)	14.90 (±2.60)	17.40 (±4.582)	0.352	
Blood sugar	0.70-1.15g/L	4.37 (±1.456)	4.24 (±1.076)	4.59 (±1.064)		
HbA1c	4.5-6.5%	9.90 (±4.384)	9.70 (±1.386)	10.88 (±2,832)	0.763	
Creatinine	2.4-8.7 mg/L (21-77mmol/L)	8.25 (±3.040)	10.88 (±6.896)	11.23 (±10.364)	0.401	
Urea	0.15-0.38 g/L	0.27 (±0.106)	0.33 (±0.120)	0.37 (±0.323)	0.013	
Sodium ion	135-145 mmol/L	155.50 (±17.677)	120 (±13.625)	13.62 (±20.083)	0.008	
Potassium ion	3.5-5.1 mmol/L	3.26 (±1.07)	2.89 (±0.423)	3,01 (±0.750)	0.003	
Chloride ion	95-105 mmol/L	114 (±1.414)	120.84 (±18.201)	118.89 (±23.534)	0.359	
Glycosuria	<2.50 g/L	15 (±7.07)	10 (±5)	(±5.36)	0.300	
Ketonuria	<0.15 g/L	0.4750 (±459)	0.4786 (±0.237)	0.4808 (±0.309)	0.112	
Proteinuria	/	2 (±.000)	1.86 (±0.378)	1.90 (±0.298)	0.126	
GFR (mL/min/1.73m²)	>60 mL/min/1.73m²	55.99 (±22.775)	61.44 (±39.01)	78.56 (±33.07)	0.011	

The Chi-square test allowed us to compare the abnormalities of biomarkers of renal function according to glycemic control, which itself is determined by the level of HbA1c (Table 5). This table shows that the high level of ketone bodies in the urine is more frequent in type 1 diabetic children with poor glycemic control compared to those with good glycemic control. It is the same trend with glycosuria, however, with different distributions that can be observed in this table. Hypercreatinine is higher in children with poor glycemic control than in those with good control with percentages of 49.1 versus 25. End-stage and early renal failure are 1.9% and 32.2% in children with poor glycemic control while low percentages were observed in those with good control. Hyperuremia also has the same tendencies as hypercreatinine. As far as ions are concerned, the values are more or less in favor of an increase in chlorine or a decrease in sodium in the blood level. Moreover, no distribution of the variables between the two groups was significant.

**Table 5:** Distribution of renal parameters according to glycemic control GFR, glomerular filtration rate

Variables	Abnormalities	HbA1c < 7% Good glycemic control	HbA1c > 7% Poor glycemic control	p-value
Ketonuria	Negative	11	1 (1.9)	0.274
	1 cross	3 (37.5)	11 (20.8)	
	2 cross	1 (12.5)	23 (43.4)	
	3 cross	3 (37.5)	17 (32.1)	
	4 cross	0 (0)	1 (1.9)	
Glycosuria	Negative	0 (0)	1 (1.9)	0.407
	1 cross	1 (12.5)	2 (3.8)	
	2 cross	1 (12.5)	13 (24.5)	
	3 cross	3(37.5)	29 (54.7)	
	4 cross	3(37.5)	8 (15.1)	
Creatinine	Hypercreatinemia	2(25)	26 (49.1)	0.203
	Normal	6 (75)	27 (50.9)	
GFR	End-stage renal faillure	0(0)	1(1.9)	0.876
	Beginning renal failure	2(25)	16 (32.2)	
	Normal range	6(75)	36(67.9)	
Urea	Hypouremia	2 (25)	10(18.9)	0.910
	Normal	3(37.5)	20 (37.7)	
	Hyperuremia	3 (37.5)	23 (43.4)	
Potassium ion	Hypokalemia	4 (50)	43 (81.1)	0.051
	Normal	4 (50)	10 (18.9)	
Chloride ion	Hypochloremia	0 (0)	3 (5.7)	0.755
	Normal	2 (25)	15 (28.3)	
	Hyperchloremia	6 (75)	35 (66)	
Sodium ion	Hypernatremia	5 (62.5)	34 (64.2)	0.816
	Normal	1 (12.5)	10 (18.9)	
	Hypernatremia	2 (25)	9 (17)	

The ANOVA test permitted the distribution of the means between the two groups in Table 6. The mean of ketonuria is 0.4063 against 0.4915, respectively, in children with good control (HbA1c<7%) and those with poor control (HbA1c>7%). The average glycosuria in children with good control is higher than that of the group with poor control. The same is true for GFR, potassium, and sodium. The variation of these averages is opposite for the case of urea and chlorine. The p-values are in no way significant for all the parameters evaluated.

**Table 6:** Distribution of average kidney parameters according to glycemic control GFR, glomerular filtration rate

Variables	Usual values	HbA1c < 7%	HbA1c>7%	P-value
Ketonuria	<0.15 g/L	0.4063±0.34376	0.4915±0.29625	0.84
Glycosuria	<2.50 g/L	12.1875±6.99968	9.8113±5.06802	0.76
Creatinine	2.4-8.7 mg/L	9.2400±7.78702	11.3753±10.12852	0.771
GFR	>60 mL/min/1.73m²	86.0338±37.97928	74.3668±33.09035	0.713
Urea	0.15-0.38 g/L	0.3088±0.18597	0.3736±0.31519	0.759
Potassium ion	3.5-5.1 mmol/L	3.4963±0.42735	2.9368±0.73060	0.186
Chloride ion	95-105 mmol/L	112.7750±13.43022	119.8951±23.49188	0.079
Sodium ion	135-145 mmol/L	135.5250±21.67103	130.9453±19.93500	0.960

Values of different parameters of the study according to the level of HbA1c and glycemic control marker are listed in Table 7. For this study, all patients were on treatment and not only patients treated with insulin were included but also those taking oral antidiabetics (OAD). 93.4% of patients were on insulin, 3.3% were on oral antidiabetics, and 3.3% were taking both insulin and oral antidiabetics. While 93.4% of our study population reported having a glucometer for self-monitoring of their blood sugar, 6.6% did not.

**Table 7:** Overall distribution of patients according to parameters influencing glycemic control

Parameters	Category	HbA1c≤7 (%)	HbA1c>7 (%)	P-value
Educational level	Not in school	3 (37.5)	16 (69.80)	0.84
	Primary	2 (25)	20 (37.73)	
	Secondary	3 (36.5)	17 (32.07)	
Parent function	Unemployed	1 (12.5)	11 (79.23)	0.22
	Informal sector	4 (50)	19 (35.84)	
	Official	3 (37.5)	23 (43.39)	
Current residence	Urban	7 (87.5)	42 (79.24)	0.66
	Rural	1 (12.5)	11 (20.76)	
Age group	0-5 years	1 (12.5)	1 (1.89)	0.17
	6-10 years	0	7 (13.20)	
	11-17 years	7 (87.5)	45 (84.90)	
Sex	Male	7 (87.5)	27 (50.90)	0.05
	Feminine	1 (12.5)	26 (49.09)	
Duration of diabetes	Inaugural (less than 1 month)	6 (75%)	44 (83.01%)	0.850
	1-12 Months	1 (12.5%)	5 (9.43%)	
	>12 Months	1 (12.5%)	3 (7.55%)	
Possession of the glucometer	Yes	8 (100%)	49 (92.45%)	0.431
	No	0	4 (7.54%)	
Treatment	Insulin	8 (100%)	49 (92.45%)	0.724
	Oral antidiabetics	0	2 (3.77%)	
	Insulin and oral antidiabetics	0	2 (3.77%)	
Tachycardia	Yes	6 (75%)	52 (98.11%)	0.005
	No	2 (25%)	1 (1.88%)	
Oral antidiabetics	None	8 (100%)	45 (84.90%)	0.605
	Metformin	0	5 (9.43%)	
	Metformin+Sulfamide	0	1 (5.66%)	
Cow milk	Yes	1 (12.5%)	13 (24.53%)	0.451
	No	7 (87.5%)	40 (75.47%)	
Pubertal state	Pubertal state	4 (50%)	22 (41.50%)	0.201
	Normal puberty	4 (50%)	16 (30.18%)	
	Puberty delay	0	15 (28.31%)	

Since puberty is a parameter influencing glycemic control, 42.6% of patients were unaware of their puberty status, 32.8% had normal puberty, and 24.6% had delayed puberty. It should be noted that despite all this, 95.1% of children had tachycardia. The survey sheets revealed that only 23% consume cow's milk. This is shown in Table 7.

The population with the highest poor glycemic control was that of children whose diabetes is inaugural (just discovered). As the duration of the discovery of diabetes increased, poor glycemic control decreased.

The Chi-square test permitted to distribute the renal biochemical abnormalities according to the duration of diabetes in Table 8. From this table, it appears that the prevalence of terminal renal failure is 2% in the group of children with inaugural diabetes against 0% in the other groups. Early renal failure is also high in children with an inaugural duration of diabetes with 15% against low numbers and percentages in other groups. The same observations were made for other anomalies such as hypercreatinemia, hyperuremia, hyperchloremia, hyperglycosuria, and hyperketonuria. Abnormalities such as hyponatremia and hypokalemia were also more prevalent in children with onset diabetes. All these results show that abnormalities in renal biochemical parameters are more frequent in diabetic children with inaugural duration than in other diabetic children.

**Table 8:** Distribution of biochemical abnormalities according to the duration of diabetes RF, Renal failure; GFR, glomerular filtration rate

Variables	Abnormalities	Duration of the diabetes				P-value
		Inaugural (less than 1 month)	1 to 12 months	13 to 24 months	25 to 48 months	
GFR	Terminal RF	1 (2%)	0	0	0	0.787
	Beginning RF	15 (30%)	1 (16.7%)	1 (25%)	1 (100%)	
	Normal GFR	34 (68%)	5 (83.3%)	3 (75%)	0	
Creatinine	Normal	26 (52%)	4 (66.7%)	3 (75%)	0	0.503
	Hyper	24 (48%)	2 (33.3%)	1 (25%)	1 (100%)	
Urea	Hypo	10 (20%)	1 (16.7%)	1 (25%)	0	0.913
	Normal	19 (38%)	2 (33.3%)	1 (25%)	1 (100%)	
	Hyper	21 (42%)	3 (50%)	3 (50%)	0	
Sodium ion	Hypo	33 (66%)	3 (50%)	2 (50%)	1 (100%)	0.454
	Normal	10 (20%)	0	1 (25%)	0	
	Hyper	7 (14%)	3 (50%)	1 (25%)	0	
Chloride ion	Hypo	3 (6%)	0	0	0	0.728
	Normal	12 (24%)	3 (50%)	2 (50%)	0	
	Hyper	35 (70%)	3 (50%)	2 (50%)	1 (100%)	
Potassium ion	Hypo	38 (80.9%)	5 (83.3%)	3 (75%)	1 (100%)	0.925
	Normal	12 (11.5%)	1 (16.7%)	1 (25%)	0	
Glycosuria	Negative	1 (2%)	0	0	0	0.002
	1 cross	0	1 (16.7%)	1 (25%)	1 (100%)	
	2 cross	11 (22%)	2 (33.3%)	1 (25%)	0	
	3 cross	29 (58%)	1 (16.7%)	2 (50%)	0	
	4 cross	9 (18%)	2 (33.3%)	0	0	
Ketonuria	Negative	1 (2%)	0	1 (25%)	0	0.264
	1 cross	9 (18%)	3 (50%)	1 (25%)	1 (100%)	
	2 cross	20 (40%)	2 (33.3%)	2 (50%)		
	3 cross	19 (38%)	1 (16.7%)	0		
	4 cross	1 (2%)	0	0		

The average of the renal parameters is also divided according to the duration of the diabetes (Table 9). The observation in this table shows that parameters like ketone bodies gradually decrease as the diabetes lasts. The children with inaugural duration have an average ketone body of 0.5230, those with a duration of six to 12 months have an average of 0.3417, those with a duration of 18 to 24 months have an average of 0.2375, and finally, those from 42 to 48 have an average of 0.1500. These trends are the same with glycosuria with respective values of 10.5000, 10.4167, 6.8750, and 2.5000 according to the gradual duration of diabetes as shown in the table. However, the other renal parameters showed no significant differences.

**Table 9:** Distribution of mean renal parameters according to duration of diabetes GFR, glomerular filtration rate

Parameters	Usual values	Inaugural	6 to 12 months	12 to 24 months	24 to 48 months	P-value
Cetonic corpse	<0.15 g/L	0.5230±0.30122	0.3417±0.25577	0.2375±0.19738	0.1500±0	0.375
Glycosuria	<2.50 g/L	10.5000±5.07595	10.4167±7.81292	6.8750±3.75000	2.5000±0	0.163
Creatinine	2.4-8.7 mg/L (21-77 mmol/L)	11.4190±10.58507	8.5767±3.45006	8.8500±6.19328	19.0000±0	0.576
GFR	2.4-8.7 mg/L (21-77 mmol/L)	74.1298±31.91769	85.4650±34.40945	96.5250±48.56856	24.3300±0	0.713
Uremia	0.15-0.38 g/L	0.3724±0.32382	0.3400±0.14297	0.3725±0.20839	0.1200±0	0.721
Potassium ion	3.5-5.1 mmol/L	3.0388±0.71974	2.7667±0.79099	3.0400±0.87377	2.9200±0	0.915
Chloride ion	95-105 mmol/L	121.2338±23.09461	105.5250±14.4407	104.2000±9.01406	145.0000±0	0.167
Sodium ion	135-145 mmol/L	130.3860±19.85846	141.500±18.61988	134.0000±27.21519	120.0000±0	0.842

Descriptive statistics using the Chi-square test show the results of the p-value of the parameters as a function of the GFR (Table 10). From this table, it appears that age, potassium ion, ketonuria, and cow milk are associated with GFR.

**Table 10:** Correlation between GFR and other parameters **The correlation is significant at the 0.01 level (two-sided). OAD, oral antidiabetics; GFR, glomerular filtration rate; BMI, body mass index

GFR		
Parameters	P-value	X²
Age	0.006^**^	19.486
Sex	0.329	2.226
BMI	0.59	1.668
Duration of diabetes	0.15	0.070
HbA1c	0.50	0.265
Urea	0.088	7.942
Sodium ion	0.088	2.609
Potassium ion	0.001^**^	2.475
Chloride ion	0.52	0.861
Glycosuria	0.87	3.993
Ketonuria	0.019^**^	5.137
Proteinuria	0.71	0.147
Cow milk	0.004^**^	5.033
Pubertal state	0.08	7.603
Infections	0.26	4.544
OAD	0.59	0.792
Ketoacidosis	0.70	0.412

The Spearman correlation test carried out shows positive and negative associations with significant differences between the parameters, in particular between the level of creatinine and urea with a p-value of 0.01 and between the GFR and creatinine with a p-value of 0.01 (Table 11), respectively, positive and negative. A negative association between the duration of diabetes and the rate of ketone bodies with p=0.05 and between the BMI and the chloride ion (Table 11) was also observed. These correlations are listed in the table.

**Table 11:** Spearman correlation between the different parameters **The correlation is significant at the 0.01 level (two-sided). *The correlation is significant at the 0.05 level (two-sided). BMI, body mass index; GFR, glomerular filtration rate; OAD, oral antidiabetics

Parameters	Duration of the diabetes	BMI	HbA1c	Glycosuria	Proteinuria	GFR	Creatinine	Urea	Ketones	K	Cl	Na	OAD	Age range
Duration of the diabetes	1.000	-0.025	-0.089	-0.226	0.155	0.048	-0.095	0.015	-0.335^**^	-0.056	-0.096	0.128	-0.003	0.194
BMI	-0.025	1.000	-0.260^*^	-0.037	-0.131	0.041	-0.113	-0.085	-0.134	0.088	-0.106	-0.204	0.326^*^	0.171
HbA1c	-0.089	-0.260^*^	1.000	-0.115	-0.128	-0.055	0.163	0.052	0.111	-0.250	-0.074	-0.029	0.128	-0.009
Glycosuria	-0.226	-0.037	-0.115	1.000	-0.061	-0.030	0.161	-0.050	.672^**^	0.104	0.288^*^	-0.157	-0.280^*^	-0.091
Proteinuria	0.155	-0.131	-0.128	-0.061	1.000	0.012	-0.027	0.094	-0.116	0.180	0.103	0.242	-0.073	0.013
GFR	0.048	0.041	-0.055	-0.030	0.012	1.000	-0.728^**^	-0.331^**^	0.140	0.201	0.048	-0.040	-0.008	0.208
Creatinine	-0.095	-0.113	0.163	0.161	-0.027	-0.728^**^		0.352^**^	0.054	-0.112	-0.111	-0.116	0.130	-0.079
Urea	0.015	-0.085	0.052	-0.050	0.094	-0.331^**^	0.352^**^	1.000	-0.189	-0.025	-0.343^**^	0.181	-0.234	0.092
Ketone	-0.335^**^	-0.134	0.111	0.672^**^	-0.116	0.140	0.054	-0.189	1.000	-0.047	0.104	-0.297^*^	-0.186	-0.014
K	-0.056	0.088	-0.250	0.104	0.180	0.201	-0.112	-0.025	-0.047	1.000	0.038	0.010	-0.051	0.104
Cl	-0.096	-0.106	-0.074	0.288^*^	0.103	0.048	-0.111	-0.343^**^	0.104	0.038	1.000	-0.125	-0.108	-0.108
Na	0.128	-0.204	-0.029	-0.157	0.242	-0.040	-0.116	0.181	-0.297^*^	0.010	-0.125	1.000	-0.062	0.021
OAD	-0.003	0.326^*^	0.128	-0.280^*^	-0.073	-0.008	0.130	-0.234	-0.186	-0.051	-0.108	-0.062	1.000	0.137
Age range	0.194	0.171	-0.009	-0.091	0.013	0.208	-0.079	0.092	-0.014	0.104	-0.108	0.021	0.137	1.000

## Discussion

Diabetes is a metabolic disorder characterized by chronic hyperglycemia due to defects in the secretion and/or action of insulin that can lead to impaired renal function. The kidney is an organ for filtering and purifying the blood, a regulator of the organism. The alteration of the latter leads to renal failure and nephropathy and creates a homeostatic imbalance within the body. The general objective of our study was to evaluate the impact of type 1 diabetes on renal parameters in children aged one to 17 years at the Mother and Child University Hospital in N’Djamena, Chad, and more specifically to determine the abnormalities of renal parameters, to assess the impact of glycemic control on renal indicators, and to determine the correlation between biochemical abnormalities of renal function and the duration of discovery of diabetes in these children. The analysis of the data collected on the questionnaires provided some qualitative data associated with the biochemical parameters analyzed.

The study focused on 61 children with type 1 diabetes at the Mother and Child University Hospital in N’Djamena, Chad, and those who were newly received at the laboratory and Pediatrics 2 of the said center. It appears from the Chi-square analysis that all the children were in a situation of hyperglycemia and 93.3% of them were on insulin treatment. This situation of hyperglycemia is explained by the fact that patients are unable to metabolize glucose following the defect of insulin secretion by the Langerhans beta cells of the pancreas. Any nutritional intake rich in carbohydrates must be accompanied by an injection of synthetic insulin in order to transport the molecules of glucose to the cells. These results corroborate those of previous studies [12]. Most children were educated (68.9%) including 36.1% in primary school and 32.8% in secondary school, while 42.6% of parents of children are civil servants. The level of education of the children and the function of the parents are necessary for good monitoring and glycemic control of the child. This will prevent the child from being plunged into a ketoacidotic coma. The participants in the study were divided mainly according to glycemic control and the duration of discovery of diabetes but also according to age group.

Based on glycemic control, patients were categorized into two groups; those with good control and those with poor control distributed as follows: 13.1% of children with good control and 86.9% of those with poor glycemic control with an average of 10.70 (±2.73)% in glycated hemoglobin. The variation or alteration of renal parameters was linked to poor glycemic control. A high level of serum creatinine (49.1%), urea (43.4%), chlorine (66%), glucose, and ketone bodies in the urine (98%) was noted. The elevation of these markers indicates functional renal failure and can specify the severity of ketoacidosis and guide therapeutic management. The studies of Sanogo showed similar results in Bamako [13].

Multivariate logistic regression analysis shows increased levels of creatinine and urea. Urea is known to be a nitrogenous compound formed in the liver as an end product of protein catabolism and approximately 85% of urea is eliminated by the kidneys and the rest is excreted through the gastrointestinal tract. Creatinine, a byproduct of creatine phosphate in muscle, is eliminated entirely by the kidney. A simultaneous increase in creatinine and urea in most patients with diabetes indicates renal dysfunction. This may be related to an increased catabolic rate of proteins for energy production in the body and a decrease in GFR. This process of proteolysis is explained by the fact that in energy failure due to the non-use of blood glucose, the cells seek other sources of supply among which the proteins by their degradation can release the substrates of the gluconeogenesis and amino wastes such as urea and creatinine as described by previous studies [14]. This led to moderate and severe terminal renal failure in patients, respectively, at 1.6% and 29.5% with a p-value of 0.876. This observation was consistent with that of a previous study, which showed a decrease in GFR and an increase in insufficiency [15]. In addition, kidney damage can be explained by immune complex deposition and toxin-induced nephropathy. The Pearson correlation showed negative and significant associations between GFR and creatinine (p=0.01) but also between GFR and urea (p=0.01). These high rates are due to degradation. Hyperglycosuria (97.4%), as well as hyperketonuria (96.7%), is a function of the excessive amount of unmetabolized glucose in the blood and the rate of protein degradation for the production of energy. On the other hand, some ions were recorded decreasing in the participants; hyponatremia and hypokalaemia were strongly observed in children (63.9% and 67.2%, successively). These results are in agreement with those of Bernardor and differ from the results obtained by Konate et al. in Bamako [16,17].

According to the duration of discovery, 82% of children were those whose discovery of diabetes was inaugural. The analyses show 48% of hypercreatinemia, 50% of hyperuremia in children, 70% of hyperchloremia, 98% of hyperglycosuria, and hyperketonuria (2% IRT and 30% MRI). These levels are characteristic of a chronic kidney problem (diabetic nephropathy). In this study, the moderate, onset, or severe renal failure is 30 times higher than the terminal renal failure. This is similar to previous work [18].

Limitations of the study

An increase in the size of the population can lead to the observation of all the biochemical parameters linked to the alteration of renal function in diabetic patients.

## Conclusions

From this study, it emerges that the alteration of renal function through biochemical parameters is linked to poor glycemic control and the duration of the discovery of diabetes. These data could be exploited for health care benefits in children with type 1 diabetes.
